# Habitat fragmentation affects plant–arthropod interactions through connectivity loss and edge effects

**DOI:** 10.1002/ecy.70322

**Published:** 2026-02-17

**Authors:** Katherine A. Hulting, Thomas A. H. Smith, Nick M. Haddad

**Affiliations:** ^1^ W.K. Kellogg Biological Station Michigan State University Hickory Corners Michigan USA; ^2^ Department of Integrative Biology and Program in Ecology, Evolution, and Behavior Michigan State University East Lansing Michigan USA; ^3^ Department of Integrative Biology University of Wisconsin‐Madison Madison Wisconsin USA

**Keywords:** arthropod, habitat fragmentation, landscape connectivity, pollination, pollinator, species interactions

## Abstract

Habitat fragmentation is widespread globally, but the effects of fragmentation on populations and communities are often unclear. Because species responses to fragmentation are interdependent, examining how fragmentation alters species interactions may clarify community responses to fragmentation. In a large, replicated fragmentation experiment, we tested the effects of inter‐patch connectivity, patch‐scale edge‐to‐area ratio, and within‐patch distance from an edge on multiple co‐occurring plant–arthropod interactions and pollination rate (measured by fruit–flower ratio). Using experimentally planted populations of the sandy‐woods chaffhead *Carphephorus bellidifolius*, we measured three plant–arthropod interactions: plant–pollinator interactions, plant–florivore interactions, and plant–spider interactions (predators of pollinators). Connectivity increased pollinator visitation, highlighting the significance of connectivity for pollinator foraging. Across all arthropod groups, connectivity and edge‐to‐area ratio affected visitation more than distance from an edge. Despite the strong impact of connectivity on plant–pollinator interactions, connectivity had no effect on fruit–flower ratio, indicating that other local factors may be more significant for pollination and seed set. Taken together, we provide experimental evidence that multiple plant–arthropod interactions are altered by habitat fragmentation through connectivity loss and increased edge‐to‐area ratio. Incorporating species interactions into fragmentation research will strengthen understanding of the mechanisms by which fragmentation alters populations and communities.

## INTRODUCTION

Habitat fragmentation is widespread in ecosystems (Haddad et al., 2015; Zou et al., 2025). Despite the extensive effect of fragmentation on habitat configuration globally, debate remains concerning fragmentation effects on biodiversity (Fahrig et al., 2019; Fletcher et al., 2018). This debate has arisen in part because the multiple components of habitat fragmentation and habitat loss are often confounded (Fletcher et al., 2023), paired with the complexity of responses of interacting species in a community (Didham et al., 2012). Because the response of one species to fragmentation may depend on other species' responses, examining how fragmentation affects species interactions may elucidate the impacts of fragmentation on communities (Didham et al., 2012; Ewers & Didham, 2005; Rybicki et al., 2020). Yet, species interactions are often not considered in fragmentation research (Siegel et al., 2023), and little research has been able to parse out the relative effects of multiple components of fragmentation on species interactions.

Habitat fragmentation results in several spatial changes to habitat configuration that have the potential to impact species interactions (Zou et al., 2025). As habitat is broken apart, the structural connectivity of habitat necessarily decreases, the proportion of edge habitat in a patch increases, and the average distance from an edge decreases (Fletcher et al., 2023). These components of habitat fragmentation arise at several spatial scales and may each impact species interactions, but potentially through different mechanisms. For example, connectivity loss between patches can reduce species dispersal (Baguette et al., 2013; Resasco, 2019) and community diversity (Damschen et al., 2019; Thompson et al., 2017), which may decrease the abundance of interactions or change the composition of interacting species (Batáry et al., 2021; Kormann et al., 2016). At the same time, an increase in edge habitat alters abiotic conditions (Tuff et al., 2016), which may alter species interactions through behavioral changes (Montgomery et al., 2003) or changes in community composition at edges (Caitano et al., 2020). However, because these components of fragmentation occur simultaneously when a landscape is fragmented, most past research has been unable to disentangle their relative effects on species interactions, typically focusing on patch size as a measure of fragmentation (Siegel et al., 2023). Separating out the effects of multiple fragmentation components is important for clarifying fragmentation effects on species interactions (Valente et al., 2023).

Because species interactions are interdependent, a change in one interaction due to fragmentation has the potential to shift other species interactions due to the co‐occurrence of multiple interactions (Didham et al., 2012). Considering plant–arthropod interactions, multiple arthropod groups interact with plants concurrently, and the presence of one interaction may impact other interaction types (Murphy et al., 2016). For example, pollinator visitation to plants may be deterred by the presence of pollinator predators on plants, such as flower‐dwelling spiders that predate pollinators (Antiqueira & Romero, 2016; Benoit & Kalisz, 2020). At the same time, pollinator and spider visitation may be reduced by florivores on a plant due to a reduction in flower quality from florivory (Camurça et al., 2024; Carper et al., 2016; Soper Gorden & Adler, 2016). Although these plant–arthropod interactions are co‐occurring, responses to fragmentation may differ among arthropod groups due to differences in dispersal capability or matrix tolerance between arthropod groups (Benítez‐Malvido et al., 2016; Murphy et al., 2016). However, multiple plant–arthropod interactions are rarely considered together in fragmentation research, and the relative effects of multiple fragmentation components on these plant–arthropod interactions are rarely able to be disentangled (Benítez‐Malvido et al., 2016; Brudvig et al., 2015).

Given that plant–arthropod interactions affect plant fitness and pollination success, fragmentation effects on arthropod visitation will have implications for plant populations. Flower–arthropod interactions can have positive, negative, or neutral effects on pollination and plant reproductive output, depending on the type of interaction and interaction strength (Camurça et al., 2024). For instance, the presence of spider predators may reduce pollination success by deterring or consuming pollinators (Antiqueira et al., 2020; Antiqueira & Romero, 2016; Gonçalves‐Souza et al., 2008), but the strength of this effect depends on the density of spiders on a plant (Camurça et al., 2024; Dukas & Morse, 2005). Similarly, pollinator visitation is key for the reproductive output of insect‐pollinated plants, but other factors, like the local abundance of conspecifics that impacts the pollen amount a pollinator carries, may alter the effect of pollinator visitation on pollination (Harder, 1990; Karron et al., 1995; Knight, 2003). Accordingly, fragmentation may impact plant reproductive output indirectly through shifts in plant–arthropod interactions, or directly through effects on abiotic conditions and plant population persistence that may impact flowering and pollen availability (Aguilar et al., 2006, 2019; Brudvig et al., 2015; Hulting, Brudvig, et al., 2025). Evaluating the effects of fragmentation on pollination success will further understanding of the relationship between fragmentation, species interactions, and plant reproductive output.

We tested how multiple components of habitat fragmentation affect three co‐occurring plant–arthropod interactions using experimentally planted populations of the sandy‐woods chaffhead *Carphephorus bellidifolius*. We considered three plant–arthropod interactions that are significant for plant reproductive output: plant–pollinator interactions, plant–florivore interactions, and plant–spider interactions. In a large and replicated fragmentation experiment, we tested the effects of inter‐patch connectivity, patch‐scale edge‐to‐area ratio, and within‐patch distance from an edge on arthropod visitation and pollination rate (as measured by fruit–flower ratio). Specifically, we asked: How do connectivity, edge‐to‐area ratio, and distance from an edge affect (1) pollinator, florivore, and spider visitation to *C. bellidifolius*, and (2) *C. bellidifolius* pollination rate?

## METHODS

### Site description and experimental design

**FIGURE 1:**
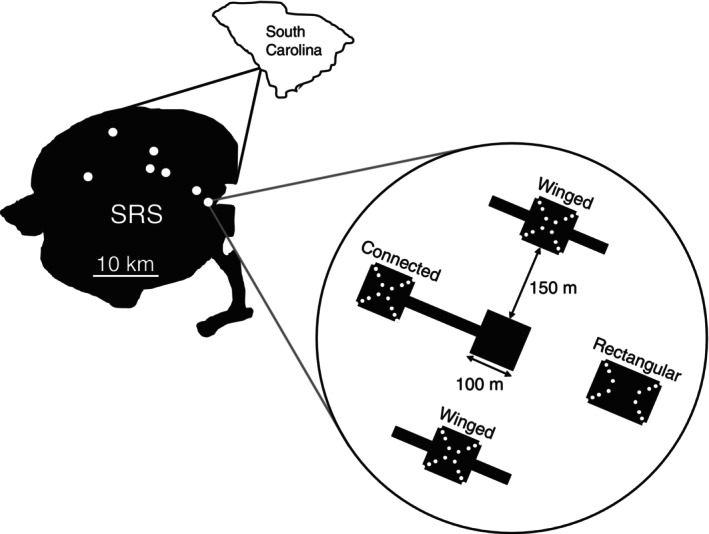
Design of experimental blocks, showing the locations of seven blocks at the Savannah River Site (SRS) in South Carolina and depiction of patches in one experimental block. Each experimental block contains five longleaf pine savanna patches, one that is connected to a center patch by a corridor (“connected”) and three that are unconnected and either high edge‐to‐area ratio (“winged”) or low edge‐to‐area ratio (“rectangular”). White points in the patches represent locations of original transplanted *Carphephorus bellidifolius* individuals, at 16 locations in each patch at 4 distances from the nearest two edges (0, 10.25, 19.10, and 36.10 m). Figure from Hulting, Brudvig, et al. (2025).

We conducted this study in seven experimentally fragmented landscapes managed by the USDA Forest Service at the Savannah River Site (SRS), a National Research Park in Aiken and Barnwell counties, South Carolina, USA. These experimental landscapes (hereafter, “blocks”) were created in 2000 (*n* = 5) and 2007 (*n* = 2) by clearing pine plantation forest to create five open patches within each block that are being restored to longleaf pine savanna through prescribed fire and periodic removal of hardwoods. All blocks contain one center 100 m × 100 m patch surrounded by four 1.375 ha peripheral patches that vary in connectivity and edge‐to‐area ratio (Figure 1). One peripheral patch is connected to the center patch by a 150 m × 25 m wide corridor (“connected patch”). The other three peripheral patches are isolated from the center patch by 150 m and are either rectangular or winged. Rectangular patches are 100 m × 137.5 m (low edge‐to‐area ratio), while winged patches are a 100 m × 100 m square with two 75 m × 25 m wings extending from opposite sides (high edge‐to‐area ratio). Four blocks contain two winged patches and one rectangular patch, while the other three contain one winged patch and two rectangular patches. The orientation and position of patches within a block are randomized. Winged and connected patches have similar edge‐to‐area ratios but differ in structural connectivity, testing for connectivity effects. Winged patches have ~50% higher edge perimeter compared to rectangular patches, testing edge‐to‐area ratio effects.

Within each block, we used experimentally planted *C. bellidifolius* individuals that are part of a long‐term population dynamics experiment (Brudvig et al., 2015; Caughlin et al., 2019; Hulting, Brudvig, et al., 2025). *C. bellidifolius* (hereafter, “*Carphephorus*”) is a perennial forb that is native to longleaf pine savanna habitat. *Carphephorus* is insect pollinated and flowers August–October, producing an average of 39.5 inflorescences per individual (Burt & Brudvig, 2019). In 2007, we propagated *Carphephorus* in greenhouses using seeds collected from other sites around SRS. In every patch, we transplanted one *Carphephorus* seedling to 16 locations at four distances from each patch corner (0, 14.5, 27, and 51 m from the patch corner, which equates to 0, 10.25, 19.10, and 36.10 m from the nearest edge) (Figure 1). To improve transplant survival, prior to transplanting, we removed vegetation surrounding each transplant location by hand‐weeding and applying a glyphosate herbicide, and within the first 12 months after transplanting, we watered in weeks that did not receive at least 2.5 cm of rainfall, which is the average for the site. We replanted dead individuals in June and October 2007, April 2008, and April 2009. *Carphephorus* occurred naturally in one patch in our experiment; prior to experimentally planting *Carphephorus*, we removed the naturally occurring individuals from this patch. However, *Carphephorus* individuals may have established from these naturally occurring individuals since removal due to persistence in the soil seed bank.

### Data collection

In September 2023, we surveyed plant–arthropod interactions using surviving and flowering *Carphephorus* plants. We aimed to survey the original transplanted *Carphephorus* individuals, and if the transplanted individual was dead or not reproductive, we surveyed a nearby *Carphephorus* recruit (*n* = 87 plants surveyed, ~20% of original plants). Surveyed plants were spaced at least 5 m apart. Due to differences in *Carphephorus* persistence and flowering between patches and blocks, the number of surveyed plants (“focal plant”) per patch type was connected (*n* = 21), rectangular (*n* = 28), and winged (*n* = 38). For each focal plant, we surveyed three plant–arthropod interactions that may be significant for plant reproductive output: plant–pollinator interactions, plant–florivore interactions, and plant–spider interactions. Specifically, we counted the number of pollinators, florivores, and spiders visiting a focal plant. Pollinators included butterflies and moths (Lepidoptera), flies (Diptera), bees (Hymenoptera: Apoidea: Anthophila), and wasps (Hymenoptera: Vespidae). Florivores included insects that are known to consume flowers or seeds prior to dispersal, such as beetles (Coleoptera, including Phalacridae, Curculionidae, and *Epicauta* spp.), katydids (Orthoptera: Tettigoniidae), and grasshoppers (Orthoptera: Acrididea) (Camurça et al., 2024; Cardel & Koptur, 2010; Theis et al., 2007). We chose to include generalist herbivores that were present on the plant inflorescences in our count of florivores, as these generalist herbivores have the potential to damage or consume flowers in addition to other parts of the plant. Spiders included sit‐and‐wait predators or actively hunting spiders on flowers, including crab spiders (Araneae: Thomisidae) and the green lynx spider (*Peucetia viridans*) (Hawn et al., 2018; Romero et al., 2011; Whitcomb et al., 1966). To survey plant–arthropod interactions, we observed each focal plant for 10 min per survey to record counts of each arthropod group, waiting until the end of the survey to collect florivores and spiders to reduce disturbance to visiting pollinators during the survey. We surveyed focal plants three times throughout September with at least 4 days between repeated surveys; however, because of variation in flowering length and timing, some focal plants were surveyed fewer than three times. An entire block was sampled in 1 day and the time of day each plant was surveyed was randomized, with all surveys between 9:30 and 17:00 on days with low wind speeds and temperatures ranging from 21 to 36°C.

Additionally, to assess pollination rate, we measured the fruit–flower ratio of *Carphephorus* inflorescences. During peak flower production, we randomly tagged up to three inflorescences per focal plant, collected them prior to seed dispersal (October–November), and stored them in the freezer. Since each *Carphephorus* flower produces an achene regardless of pollination success, we assessed the fruit–flower ratio using collected seed structures (Brudvig et al., 2015). For each inflorescence, we dissected all achenes to determine if they contained a developed seed. Achenes with signs of seed predation were counted as developed to correct for pre‐dispersal seed predation (Brudvig et al., 2015). We calculated the fruit–flower ratio as the proportion of developed seeds to the total number of achenes.

Because floral abundance may impact arthropod visitation and pollination success, we took three measures of the floral abundance: the number of focal plant inflorescences (individual plant scale), surrounding local floral abundance (community level, 5‐m scale), and full‐patch *Carphephorus* flowering abundance (population level, patch scale). First, we counted the number of inflorescences on each focal plant at the time of each survey. Next, we measured local floral abundance as the floral abundance of all insect‐pollinated plants within 5 m of the focal plant. Lastly, we measured the total abundance of *Carphephorus* inflorescences in each patch because the abundance of flowering *Carphephorus* individuals in a patch may impact pollen availability and pollination success (see Appendix S1 for additional methods).

### Statistical analysis

To clarify causal assumptions and select confounder adjustment sets for estimating the direct effect of patch type (connected, rectangular, winged) and distance from an edge on floral abundance, arthropod visitation abundance, and fruit–flower ratio, we used directed acyclic graphs (DAGs). We categorized distance from an edge as “edge” (0 and 10.25 m from edge) or “interior” (19.10 and 36.10 m from edge) (results were qualitatively unchanged if all four distances were treated independently). We constructed a DAG based on hypotheses outlined in Appendix S1: Table S2 and used the DAG to identify covariates needed to estimate the direct effect of either patch type, distance from an edge, or the combination of patch type and distance from an edge on the response variable of interest (Appendix S1: Figure S1). However, we note that the term “direct effect” is only meaningful here in the context of the other variables measured; for example, the “direct effects” of distance from an edge could be mediated through other unmeasured variables, such as sunlight or temperature (Correia et al., 2025).

First, we tested the effects of patch type and distance from an edge on our three scales of floral abundance. When constructing our DAG, we hypothesized that our plant‐scale measure (number of inflorescences) would only be affected by distance to an edge, not any direct effect from patch type. In contrast, we expected that our local‐scale measure (community floral abundance) may be affected by both patch type and distance from an edge, while our patch‐scale measure (*Carphephorus* flowering abundance) would only be affected by patch type (see Appendix S1: Table S2 for detailed hypotheses). For each scale of floral abundance, we fit one model testing these hypotheses (see Appendix S1: Table S3 for model list).

**FIGURE 2:**
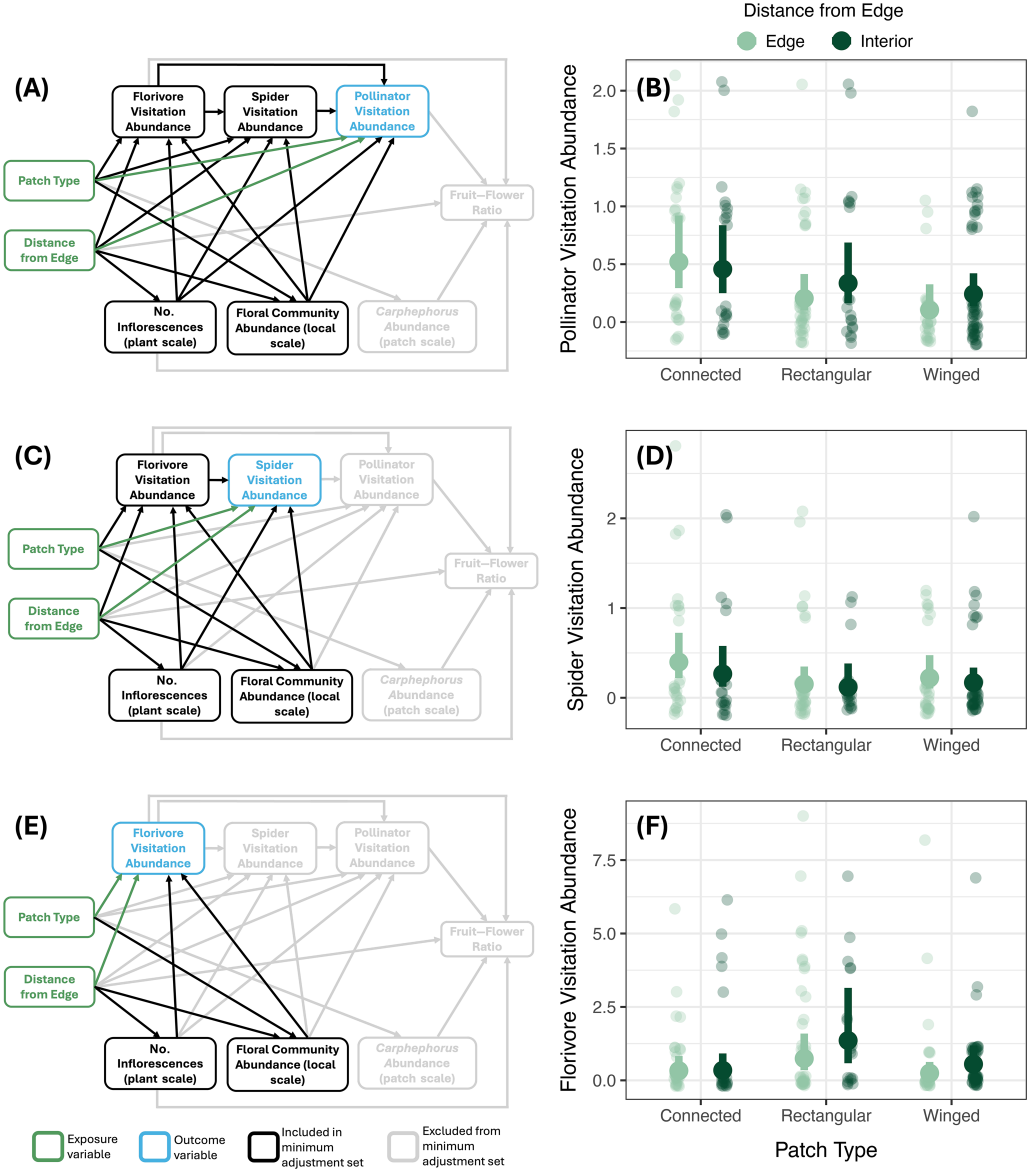
(a, c, e) Directed acyclic graphs (DAGs) to determine covariates to estimate the direct interactive effect of patch type and distance from an edge on arthropod visitation. For simplicity, the interaction between patch type and distance from an edge is not represented in the DAG. Green boxes indicate exposure variable(s), blue boxes indicate the response variable of interest, and black boxes indicate variables that were included as covariates in the model. (b) The number of pollinator visits to *Carphephorus bellidifolius* increased in connected patches compared to other unconnected patch types at the edge of patches. (d) The number of spider visits to *C. bellidifolius* did not significantly differ between patch types and edge or interior plants. (f) The number of florivore visits to *C. bellidifolius* increased in unconnected low edge‐to area rectangular patches compared to other patch types (high edge‐to‐area ratio). For all plots, solid points and error bars represent model predictions and 95% CIs, respectively. Smaller points represent the number of visits to a focal plant in one survey. Significant differences are in Appendix S1: Tables S6 and S7.

Next, we tested the effects of patch type and distance from an edge on the number of pollinator, florivore, and spider visits to *Carphephorus*. Although other measures of arthropod visitation, such as visiting species richness and community composition, are also significant for understanding species interactions, visitation abundance and richness were highly correlated due to overall low visitation rates during surveys. Because of this high correlation, we chose to investigate visitation abundance as our measure of arthropod visitation. For each arthropod group, we used our DAG to identify adjustment sets to include in our model, in addition to our predictor variables of interest (patch type, distance from an edge, and the interaction between patch type and distance from an edge). Our adjustment sets for each arthropod group are listed in Appendix S1: Table S3 and visualized in Figure 2a,c,e.

Lastly, we used our DAG to identify adjustment sets for fruit–flower ratio as a measure of pollination rate. Because we hypothesized that patch type would only impact fruit–flower ratio indirectly through other variables (arthropod visitation and floral abundance), we excluded patch type from the model. Additionally, fruit–flower ratio was measured at a different temporal scale than other variables (fruit–flower ratio measured at one time point versus repeated surveys of arthropods and floral community), so we averaged arthropod and floral measures for each focal plant to include in our fruit–flower ratio model.

We used generalized linear mixed‐effects models (GLMMs) to fit models for floral abundance, arthropod visitation, and fruit–flower ratio. We chose to use GLMMs informed by DAGs rather than a structural equation modeling (SEM) approach because the structure of our data limited our ability to fit a full SEM based on our hypotheses. We used a negative binomial distribution to model the number of inflorescences (plant scale), and pollinator, spider, and florivore visitation abundance, a Gaussian distribution to model community floral abundance (local scale) and *Carphephorus* flowering abundance (patch scale), and a beta‐binomial distribution (weighted by the number of flowers per inflorescence) to model fruit–flower ratio. We tested for significance of fixed effects using Wald χ^2^ type III tests, and if patch type was a significant predictor, we used Tukey post hoc tests to evaluate differences between patch types. The unique ID of each focal plant (*n* = 87) and that of the experimental block (*n* = 7) were included as nested random intercepts for each model. We did not include sampling round as a random intercept because the estimated variance was very small and including it did not improve model fit or influence results.

All analyses were conducted in R v. 4.2.3 (R Core Team, 2024). We used the dagitty package v. 0.3‐4 to construct DAGs (Textor et al., 2016), the glmmTMB package v. 1.1.7 to fit generalized linear models (Brooks et al., 2017), tested for model suitability using the DHARMa package v. 0.4.6 (Hartig, 2022) and performance package v. 0.10.3 (Lüdecke et al., 2021), and tested for significance using the car package v. 3.1‐2 (Fox & Weisberg, 2019) and emmeans package v. 1.8.5 (Lenth, 2023). All figures were created with ggplot v. 3.4.2 (Wickham, 2016) and ggeffects v. 1.2.1 (Lüdecke, 2018).

## RESULTS

### Floral abundance

The number of focal plant inflorescences (individual plant scale) did not differ between the edge and interior of patches (Appendix S1: Figure S2a, Tables S4 and S5). Community floral abundance (local scale), however, decreased by 13% (95% CI: 6%, 21%) in edge plots compared to interior plots, except in rectangular patches where flowering abundance was not different between the edge and interior (Appendix S1: Figure S2d, Tables S4 and S5). *Carphephorus* flowering abundance (patch scale) was not affected by patch type (Figure 2f; Appendix S1: Tables S4 and S5).

### Plant–arthropod interactions

We found that pollinator visitation to *Carphephorus* decreased by 61% (95% CI: 8%, 83%) in unconnected rectangular and decreased by 80% (95% CI: 30%, 94%) in unconnected winged patches compared to connected patches, but only at focal plants at the edge (Figure 2b, Appendix S1: Tables S4 and S6). Pollinator visitation, however, did not differ between patch types at focal plants located in the interior of patches (Appendix S1: Tables S4 and S7). Pollinator visitation increased strongly with the number of inflorescences on the focal plant but was not affected by local floral abundance or other arthropod groups (Appendix S1: Table S4). The most common pollinators visiting *Carphephorus* were butterflies (56% of visits) followed by flies (26% of visits). Pollinator visitation to *Carphephorus* was generally low, with 0–2 visits per survey.

Spider visitation was not affected by patch type or distance to an edge (Figure 2d, Appendix S1: Tables S4 and S6). Spider visitation marginally increased with the number of inflorescences on the focal plant but was not affected by the visitation of florivores or surrounding floral abundance (Appendix S1: Table S4). Spider visitation to *Carphephorus* ranged from 0 to 3 visits per survey.

Florivore visitation to *Carphephorus* decreased by 82% (95% CI: 29%, 95%) in the interior of connected patches compared to the interior of rectangular patches (Figure 2f, Appendix S1: Tables S4 and S6). Florivore visitation decreased in winged patches compared to rectangular patches as well, although this effect was not significant (Appendix S1: Table S6). Florivore visitation was not affected by the number of inflorescences on the focal plant but decreased with higher local floral abundance (Appendix S1: Table S4). The most common florivores on *Carphephorus* were beetles, including flower beetles, blister beetles, and weevils. Florivore visitation abundance to *Carphephorus* ranged from 0 to 9 visits per survey.

### Pollination success

**FIGURE 3:**
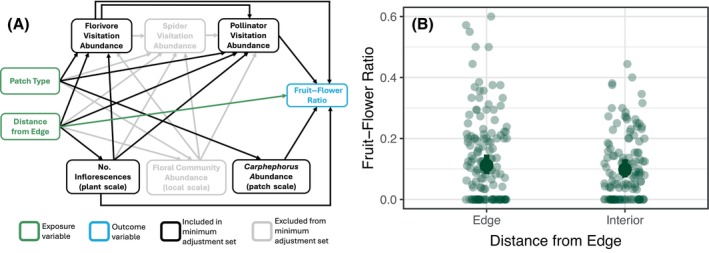
(a) Directed acyclic graphs (DAGs) to determine covariates to estimate effects on fruit–flower ratio. Green boxes indicate exposure variable(s), blue boxes indicate the response variable of interest, and black boxes indicate variables that were included as covariates in the model. (b) Fruit–flower ratio was not affected by distance from an edge. Solid points and error bars represent model predictions and 95% CIs, respectively. Smaller points represent the fruit–flower ratio of one *Carphephorus bellidifolius* inflorescence. Results are in Appendix S1: Tables S4, S6, and S7.

Arthropod visitation and distance from an edge did not affect fruit–flower ratio (Figure 3, Appendix S1: Table S4). Fruit–flower ratio decreased with a higher number of inflorescences on the focal plant and increased with a higher abundance of *Carphephorus* at the patch scale, but both effect sizes were small (Appendix S1: Table S4).

## DISCUSSION

Our results provide experimental evidence that inter‐patch connectivity and patch edge‐to‐area ratio impact multiple plant–arthropod interactions. We show that connectivity between patches increased pollinator visitation to *C. bellidifolius*, a plant species of conservation interest. Florivore visitation was typically higher in patches with low edge‐to‐area ratio, but spider visitation was not affected by connectivity or edge‐to‐area ratio, indicating that the effects of fragmentation may differ among arthropod groups. However, despite the varying effects of connectivity and edge‐to‐area ratio on plant–arthropod interactions, these changes to arthropod visitation did not affect pollination rates, as measured by fruit–flower ratio. Accordingly, habitat fragmentation may alter plant–arthropod interactions by disrupting connectivity and increasing edge‐to‐area ratio, but other factors may be significant for determining pollination success and plant reproductive output, such as pollen quality or availability.

The increase in pollinator visitation to *Carphephorus* with connectivity demonstrates the important effect of connectivity for pollinators. Previous research has demonstrated that connectivity increases pollinator dispersal (Van Geert et al., 2010), colonization (Griffin & Haddad, 2021), and biodiversity (Brückmann et al., 2010; Rotchés‐Ribalta et al., 2018), and our research shows that the benefits of connectivity also extend to pollinator interactions. Connectivity may increase pollinator visitation to plants by increasing pollinator access to resources in multiple patches, which may increase the abundance of pollinators dispersing between connected patches (Boscolo et al., 2017). Although pollinator visitation was lower in unconnected patches compared to connected patches overall, this effect was not significant at interior parts of patches. Visitation in unconnected rectangular and unconnected winged patches tended to be slightly higher at the interior of the patch, indicating that a reduction in pollinator visitation in unconnected patches primarily occurs at the edge. These results emphasize the significance of maintaining and restoring connectivity to promote foraging through plant–pollinator interactions.

Our finding that a high edge‐to‐area ratio decreased plant–florivore interactions complements previous research that has found that insect herbivory is often decreased by high edge‐to‐area ratio patches (Evans et al., 2012; Haynes & Crist, 2009; Levey et al., 2016). Increased proportion of edge habitat in a patch can alter several abiotic conditions, such as shifting average temperatures or moisture availability (Tuff et al., 2016). In our system, with open habitat patches and a forested matrix, patches with low edge‐to‐area ratio tend to have a higher proportion of habitat that is warmer, which may have increased the density of florivores in the patch (Evans et al., 2012). Our results suggest that patches with low edge amounts may increase florivore visitation, which may subsequently lead to increased florivory.

Although connectivity and edge‐to‐area ratio influenced pollinator and florivore visitation, we found little consistent effect of distance from an edge on arthropod visitation. Because edges are shadier and cooler due to canopy cover in our experiment (Evans et al., 2012), we anticipated that pollinators would be deterred from visiting *Carphephorus* individuals near edges, which would subsequently reduce spider predators (Balogun et al., 2022; Watson et al., 2022). While the effect of connectivity and edge‐to‐area ratio occasionally strengthened at varying distances from the edge, distance from an edge within a patch type did not significantly impact arthropod visitation. Community floral abundance increased in interior areas of the patch, likely due to reduced sunlight and higher amounts of leaf litter (Turley et al., 2017), but this higher floral abundance did not increase arthropod visitation. Although local floral abundance typically increases the abundance of pollinators in an area (Bruckman & Campbell, 2014; Vrdoljak et al., 2016), the lack of other floral species surrounding our focal plants near edges may have promoted visitation to *Carphephorus* as one of the few options for visitation in the local area. As such, *Carphephorus* individuals at both edge and interior locations may have received similar arthropod visitation due to reduced competition for arthropod visits among floral resources at the edge of the patch. Additionally, other measures of visitation that we were unable to measure, such as visiting species richness or composition, may have been affected by edge even if visitation abundance was not.

Despite changes in plant–arthropod interactions, we did not find any effect of arthropod visitation on pollination rate, as measured by fruit–flower ratio. Given the increase in pollinator visitation to *Carphephorus* in connected patches, we expected the fruit–flower ratio to increase as well. However, the fruit–flower ratio increased slightly in patches with a higher total *Carphephorus* flowering abundance, indicating that within‐patch pollen availability may be more important for pollination success than pollinator visits alone. Because patch‐scale *Carphephorus* flowering abundance was not affected by patch type, this may have led to similar pollination rates across patches, even with changing arthropod visitation. Although pollinator visitation increased in connected patches, increased visitation may only be meaningful for pollination success if plants are pollen limited. In our experiment, where patches often contain multiple flowering individuals of *Carphephorus*, this increase in pollinator visitation may not have been as important for pollination success. Additionally, although connectivity may increase pollen movement through pollinator dispersal (Kormann et al., 2016; Townsend & Levey, 2005), our measure of fruit–flower ratio is based on the proportion of developed seeds, which may be influenced by factors such as pollen and flower quality in addition to pollen movement (Aizen & Harder, 2007; Fowler et al., 2016; Thomson et al., 2012).

Understanding how fragmentation affects species interactions is critical for understanding how landscape changes impact ecological systems and implementing strategies for biodiversity conservation (Didham et al., 2012; Hagen et al., 2012; Siegel et al., 2023). We experimentally demonstrate that habitat fragmentation, through connectivity loss and edge effects, alters multiple plant–arthropod interactions, which may lead to impacts on both plant and arthropod persistence in fragmented areas. Importantly, connectivity increased pollinator visitation to plants, highlighting the significance of maintaining connectivity for pollinator foraging. Our findings suggest that multiple components of fragmentation, each occurring at different spatial scales, may alter species interactions in ecosystems. As a result, parsing out the relative impacts of fragmentation on interdependent species interactions will advance our understanding of conservation in fragmented landscapes.

## AUTHOR CONTRIBUTIONS

Katherine Hulting and Nick Haddad conceived the ideas and designed methodology; Katherine Hulting and Thomas Smith collected the data. Katherine Hulting analyzed the data and wrote the manuscript. All authors contributed critically to the drafts and gave approval for publication.

## CONFLICT OF INTEREST STATEMENT

The authors declare no conflicts of interest.

## Supporting information


Appendix S1.

## Data Availability

Data (Hulting, Smith, & Haddad, 2025) are available in Dryad at https://doi.org/10.5061/dryad.w9ghx3g48. Code (Hulting, 2025) is available in Zenodo at https://doi.org/10.5281/zenodo.17780581.
