# Supplementary material for: Habitat fragmentation affects plant–arthropod interactions through connectivity loss and edge effects
Source: Ecology. 2026 Feb 17;107(2):e70322. doi: 10.1002/ecy.70322 (PMC12912847; doi:10.1002/ecy.70322)
Supplement: Supplementary file 1 — Appendix S1. [file ECY-107-e70322-s001.pdf]

Appendix S1

Habitat fragmentation affects plant–arthropod interactions through connectivity loss and edge effects

Katherine A. Hulting, Thomas A.H. Smith, Nick M. Haddad

Ecology

Supplementary Methods

Local floral abundance: To measure local floral abundance, we counted all flowering individuals per species within 5m of the focal plant at the time of each survey. We counted the number of open inflorescences for up to 10 individuals per species, then multiplied the average of these 10 individuals by the total number of individuals to obtain an estimate of the number of open inflorescences per species. We then added together the estimated number of inflorescences per species to obtain an estimate of the total number of open inflorescences surrounding each focal plant.

Full-patch *Carphephorus* abundance: Because the abundance of flowering *Carphephorus* individuals in a patch may impact pollen availability and pollination success, we measured the *Carphephorus* flowering abundance of each patch. We surveyed the number of flowering *Carphephorus* inflorescences in a 5 m radius surrounding up to ten flowering focal plants in each patch, or all focal plants if fewer than ten individuals were flowering in a patch. Focal plants were at least 5 m from other surveyed plants to avoid duplicate counts. We summed the floral abundance of *Carphephorus* from these 5 m radii in each patch to obtain an estimate of full-patch *Carphephorus* floral abundance.

Table S1. Number of focal plants located in each patch replicate within a block.

Experimental block	*Patch replicate*	*Patch type*	*Number of focal plants*
10	B	Connected	6
10	C	Rectangular	1
10	D	Winged	5
10	E	Rectangular	4
52	B	Connected	3
52	C	Winged	7
52	D	Rectangular	1
52	E	Winged	5
53N	B	Connected	1
53N	C	Winged	3
53N	D	Rectangular	3
53N	E	Winged	2
53S	B	Connected	1
53S	C	Winged	3
53S	D	Rectangular	5
53S	E	Winged	2
54S	B	Connected	4
54S	C	Rectangular	6
54S	D	Winged	2
54S	E	Rectangular	2
57	B	Connected	1
57	C	Rectangular	1
57	E	Rectangular	1
8	B	Connected	5
8	C	Winged	5
8	D	Rectangular	4
8	E	Winged	4

Table S2. List of hypotheses that guided the Directed Acyclic Graph (DAG).

Predictor variable	Response variable	Hypothesis
Distance from edge (edge, interior)	Number of inflorescences (plant scale)	Previous research has found that the number of *Carphephorus* inflorescences decreases near the forested matrix edge in our experiment, likely due to increased shading from canopy cover (Hulting et al. 2025).
Distance from edge (edge, interior)	Floral community abundance (local scale)	Floral abundance may decrease near the forested matrix edge due to increased shading (Turley et al. 2017, Hulting et al. 2025).
Distance from edge (edge, interior)	Pollinator visitation abundance	Pollinator visitation abundance may decrease near the edge due to abiotic changes (increased shading and cooler temperatures) (Balogun et al. 2022, Watson et al. 2022).
Distance from edge (edge, interior)	Spider visitation abundance	Spider visitation abundance may altered near the edge due to abiotic changes (increased shading and cooler temperatures) (Delgado de la flor et al. 2024).
Distance from edge (edge, interior)	Florivore visitation abundance	Florivore visitation abundance may decrease near the edge due to abiotic changes (increased shading and cooler temperatures) (Evans et al. 2012, Lemoine et al. 2013) Previous research in our experiment found that herbivores were more abundant in warmer parts of the patch (Evans et al. 2012).
Distance from edge (edge, interior)	Fruit-flower ratio	Fruit-flower ratio may decrease near the edge due to abiotic changes (increased shading and cooler temperatures) (Kilkenny and Galloway 2008). However, previous research in our experiment found no effect of distance from an edge on *Carphephorus* pollination rate (Hulting et al. 2025)12/1/25 1:44:00 PM
Patch type (connected, rectangular, winged)	Floral community abundance (local scale)	If patch type alters species composition or relative abundance, local floral community abundance may also be affected (Uroy et al. 2019, Warneke et al. 2022). However, this effect may only be apparent further from the edge, as shading from the forested edge may

		result in similar floral abundance in all patch types.
Patch type (connected, rectangular, winged)	*Carphephorus* abundance (patch scale)	Patch types with increased edge:area ratio (connected, winged) may have decreased *Carphephorus* flowering abundance at the patch scale due to increased edge effects. Alternatively, connected patches may have increased *Carphephorus* flowering abundance if connectivity is promoting *Carphephorus* dispersal.
Patch type (connected, rectangular, winged)	Pollinator visitation abundance	Connectivity may increase pollinator visitation abundance by increasing dispersal between patches (Van Geert et al. 2010, Griffin and Haddad 2021). This effect could be moderated by distance to an edge if pollinators are deterred by edges in all patch types.
Patch type (connected, rectangular, winged)	Spider visitation abundance	Connectivity may increase spider visitation abundance by increasing dispersal and increased resource subsidies. However, previous research in our experiment found that the abundance of one flower-dwelling spider (*Peucetia viridans*) was not affected by connectivity (Hawn et al. 2018). This effect could be moderated by distance to an edge if spiders are deterred by edges in all patch types.
Patch type (connected, rectangular, winged)	Florivore visitation abundance	Patch types with high edge:area ratio (connected, winged) may decrease florivore visitation abundance due to an increase of area near the edge. Previous research in our experiment found that herbivores were more abundance in patch types with low edge:area ratio (rectangular patches) (Evans et al. 2012). This effect could be moderated by distance to an edge if florivores are deterred by edges in all patch types.
Number of inflorescences (plant scale)	Pollinator visitation abundance	A higher number of inflorescences may increase pollinator visitation to the focal plant due to increased resources and attractiveness of the plant (Ohashi and Yahara 2002, Mitchell et al. 2004)

Number of inflorescences (plant scale)	Spider visitation abundance	A higher number of inflorescences may increase spider visitation to the focal plant due to increased resources and attractiveness of the plant (Su et al. 2020)
Number of inflorescences (plant scale)	Florivore visitation abundance	A higher number of inflorescences may increase florivore visitation to the focal plant due to increased resources (Ruane et al. 2014).
Number of inflorescences (plant scale)	Fruit-flower ratio	A higher number of inflorescences may increase the likelihood of geitonogamous self pollination, which may decrease fruit-flower ratio (Ruane et al. 2014).
Floral community abundance (local scale)	Pollinator visitation abundance	Higher local floral community abundance may increase pollinator visitation due to a higher abundance of pollinators in the local area (Bruckman and Campbell 2014, Vrdoljak et al. 2016).
Floral community abundance (local scale)	Spider visitation abundance	Higher local floral community abundance may increase spider visitation due to a higher abundance of spiders in the local area (Hulting et al. 2024).
Floral community abundance (local scale)	Florivore visitation abundance	Higher local floral community abundance may increase florivore visitation due to a higher abundance of florivores in the local area (Hegland and Boeke 2006, Boaventura et al. 2022).
Carphephorus abundance (patch scale)	Fruit-flower ratio	Higher total floral abundance of *Carphephorus* in a patch may increase fruit-flower ratio due to increased pollen availability (Harder 1990, Karron et al. 1995, Knight 2003).
Florivore visitation abundance	Pollinator visitation abundance	Higher florivore visitation may decrease pollinator visitation due to a reduction in flower quality from florivores (Carper et al. 2016).
Florivore visitation abundance	Spider visitation abundance	Higher florivore visitation may decrease spider visitation due to a reduction in flower quality from florivores (Camurça et al. 2024).
Florivore visitation abundance	Fruit-flower ratio	Higher florivore visitation may decrease fruit-flower ratio due to damage to plant

		reproductive structures (McCall and Irwin 2006).
Spider visitation abundance	Pollinator visitation abundance	An increase in spider visitation may decrease spider visitation due to predator avoidance (Benoit and Kalisz 2020).
Pollinator visitation abundance	Fruit-flower ratio	Because *Carphephorus* is an insect-pollinated species, higher pollinator visitation abundance may increase fruit-flower ratio by increasing pollen movement (Burt and Brudvig 2019).

Table S3. List of variables included in models based on DAG for each response variable of interest.

Response Variable	*Exposure/Treatment Variables*	*Variables Included in Adjustment Set*
Number of inflorescences (plant-scale)	Distance from an edge	NA
Floral community abundance (local-scale)	Distance from an edge, Patch type, interaction between Distance to an edge and Patch type	NA
Carphephorus abundance (patch-scale)	Patch type	NA
Pollinator visitation abundance	Distance from an edge, Patch type, interaction between Distance to an edge and Patch type	Number of inflorescences (plant-scale), Floral community abundance (local-scale), Spider visitation abundance, Florivore visitation abundance
Spider visitation abundance	Distance from an edge, Patch type, interaction between Distance to an edge and Patch type	Number of inflorescences (plant-scale), Floral community abundance (local-scale), Florivore visitation abundance
Florivore visitation abundance	Distance from an edge, Patch type, interaction between Distance to an edge and Patch type	Number of inflorescences (plant-scale), Floral community abundance (local-scale)
Fruit-flower ratio	Distance from an edge	Number of inflorescences (plant-scale), *Carphephorus* abundance (patch-scale), Pollinator visitation abundance, Florivore visitation abundance

Table S4. Results of generalized linear mixed effects for floral abundance, plant–arthropod visitation, and fruit-flower ratio. Bolded terms indicate significant ($p < 0.05$) or marginally significant comparisons ($p < 0.10$).

Response variable	*Residual df*	*Fixed effect*	χ^2	*df*	*p-value*
Number of inflorescences (local scale)	175	Distance from edge	1.351	1	0.245
Community floral abundance (local scale)	171	**Distance from edge**	**10.223**	**1**	**0.001**
		Patch type	1.700	2	0.427
		**Patch type:Distance from edge**	**5.458**	**2**	**0.065**
Carphephorus flowering abundance (patch scale)	22	Patch type	2.917	2	0.233
Number of pollinator visits	167	Distance from edge	0.105	1	0.746
		**Patch type**	**8.735**	**2**	**0.013**
		**Number of inflorescences (plant scale)**	**13.405**	**1**	**>0.000**
		Community floral abundance (local scale)	1.557	1	0.212
		Florivore visitation abundance	0.022	1	0.883
		Spider visitation abundance	0.780	1	0.377
		Patch type:Distance from edge	2.099	1	0.350
Number of spider visits	168	Distance from edge	0.587	1	0.444
		Patch type	3.743	2	0.154
		**Number of inflorescences (plant scale)**	**3.315**	**1**	**0.069**
		Community floral abundance (local scale)	1.104	1	0.293
		Florivore visitation abundance	0.893	1	0.345
		Patch type:Distance from edge	0.032	2	0.984
Number of florivore visits	169	Distance from edge	0.057	1	0.811
		**Patch type**	**5.350**	**2**	**0.069**
		Number of inflorescences (plant scale)	0.099	1	0.753

		**Community floral abundance (local scale)**	**4.990**	**1**	**0.026**
		Patch type:Distance from edge	1.515	2	0.469
Fruit-flower ratio	242	Distance from edge	0.298	1	0.585
		**Average number of inflorescences (plant scale)**	**4.058**	**1**	**0.044**
		***Carphephorus* flowering abundance (patch scale)**	**5.922**	**1**	**0.015**
		Average number of pollinator visits	0.171	1	0.680
		Average number of florivore visits	1.998	1	0.158

Table S5. Results from pairwise comparisons of the effects of patch type or distance from an edge on floral abundance (*emmeans* post hoc). Bolded terms indicate significant ($p < 0.05$) or marginally significant comparisons ($p < 0.10$).

Response variable	*Grouping variable*	*Contrasts*	*Estimate*	*SE*	*z-ratio*	*p-value*
Number of inflorescences (local scale)	NA	Edge-Interior	-0.222	0.191	-1.162	0.245
Community floral abundance (local scale)	**Connected contrasts**	**Edge-Interior**	**-1.476**	**0.462**	**-3.197**	**0.002**
	Rectangular contrasts	Edge-Interior	-0.062	0.435	-0.143	0.886
	**Winged contrasts**	**Edge-Interior**	**-1.031**	**0.355**	**-2.907**	**0.004**
Carphephorus flowering abundance (patch scale)	NA	Connected-Rectangular	-33.47	21.7	-1.541	0.292
		Connected-Winged	-6.76	21.7	-0.311	0.948
		Rectangular-Winged	26.71	19.7	1.355	0.381

Table S6. Results from pairwise patch type comparisons of the interactive effect of patch type and distance from an edge on plant–arthropod interactions (*emmeans* post hoc). Bolded terms indicate significant ($p < 0.05$) or marginally significant comparisons ($p < 0.10$).

Response variable	*Distance from edge*	*Contrasts*	*Estimate*	*SE*	*z-ratio*	*p-value*
Number of pollinator visits	Edge contrasts	**Connected-Rectangular**	**0.935**	**0.438**	**2.136**	**0.083**
		**Connected-Winged**	**1.606**	**0.639**	**2.513**	**0.032**
		Rectangular-Winged	0.671	0.678	0.990	0.583
	Interior contrasts	Connected-Rectangular	0.308	0.449	0.686	0.772
		Connected-Winged	0.638	0.394	1.619	0.238
		Rectangular-Winged	0.329	0.422	0.781	0.715
Number of spider visits	Edge contrasts	Connected-Rectangular	0.930	0.498	1.866	0.149
		Connected-Winged	0.562	0.497	1.131	0.495
		Rectangular-Winged	-0.368	0.568	-0.648	0.794
	Interior contrasts	Connected-Rectangular	0.817	0.711	1.149	0.484
		Connected-Winged	0.445	0.531	0.839	0.679
		Rectangular-Winged	-0.372	0.688	-0.540	0.852
Number of florivore visits	Edge contrasts	Connected-Rectangular	-0.937	0.547	-1.713	0.200
		Connected-Winged	0.289	0.622	0.465	0.888
		**Rectangular-Winged**	**1.226**	**0.582**	**2.105**	**0.089**
	Interior contrasts	**Connected-Rectangular**	**-1.729**	**0.703**	**-2.459**	**0.037**
		Connected-Winged	-0.800	0.641	-1.247	0.426
		Rectangular-Winged	0.930	0.521	1.783	0.175

Table S7. Results from pairwise edge comparisons of the interactive effect of patch type and distance from an edge on plant–arthropod interactions (*emmeans* post hoc). Bolded terms indicate significant ($p < 0.05$) or marginally significant comparisons ($p < 0.10$).

Response variable	*Patch type*	*Contrasts*	*Estimate*	*SE*	*z-ratio*	*p-value*
Number of pollinator visits	Connected contrasts	Edge-Interior	0.129	0.399	0.324	0.746
	Rectangular contrasts	Edge-Interior	-0.498	0.458	-1.086	0.277
	Winged contrasts	Edge-Interior	-0.839	0.643	-1.304	0.192
Number of spider visits	Connected contrasts	Edge-Interior	0.386	0.504	0.766	0.444
	Rectangular contrasts	Edge-Interior	0.273	0.694	0.393	0.694
	Winged contrasts	Edge-Interior	0.269	0.539	0.499	0.618
Number of florivore visits	Connected contrasts	Edge-Interior	0.163	0.681	0.240	0.811
	Rectangular contrasts	Edge-Interior	-0.629	0.522	-1.205	0.228
	Winged contrasts	Edge-Interior	-0.925	0.584	-1.586	0.113

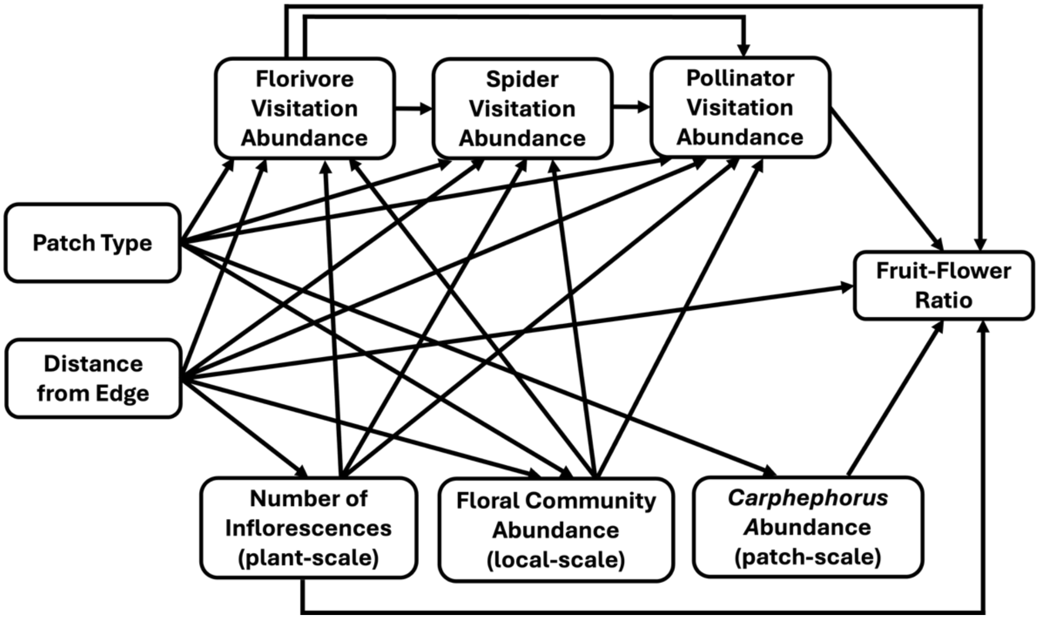

Figure S1. Directed Acyclic Graph (DAG) of expected relationships between variables. Direction of arrow indicated expected direction of relationship.

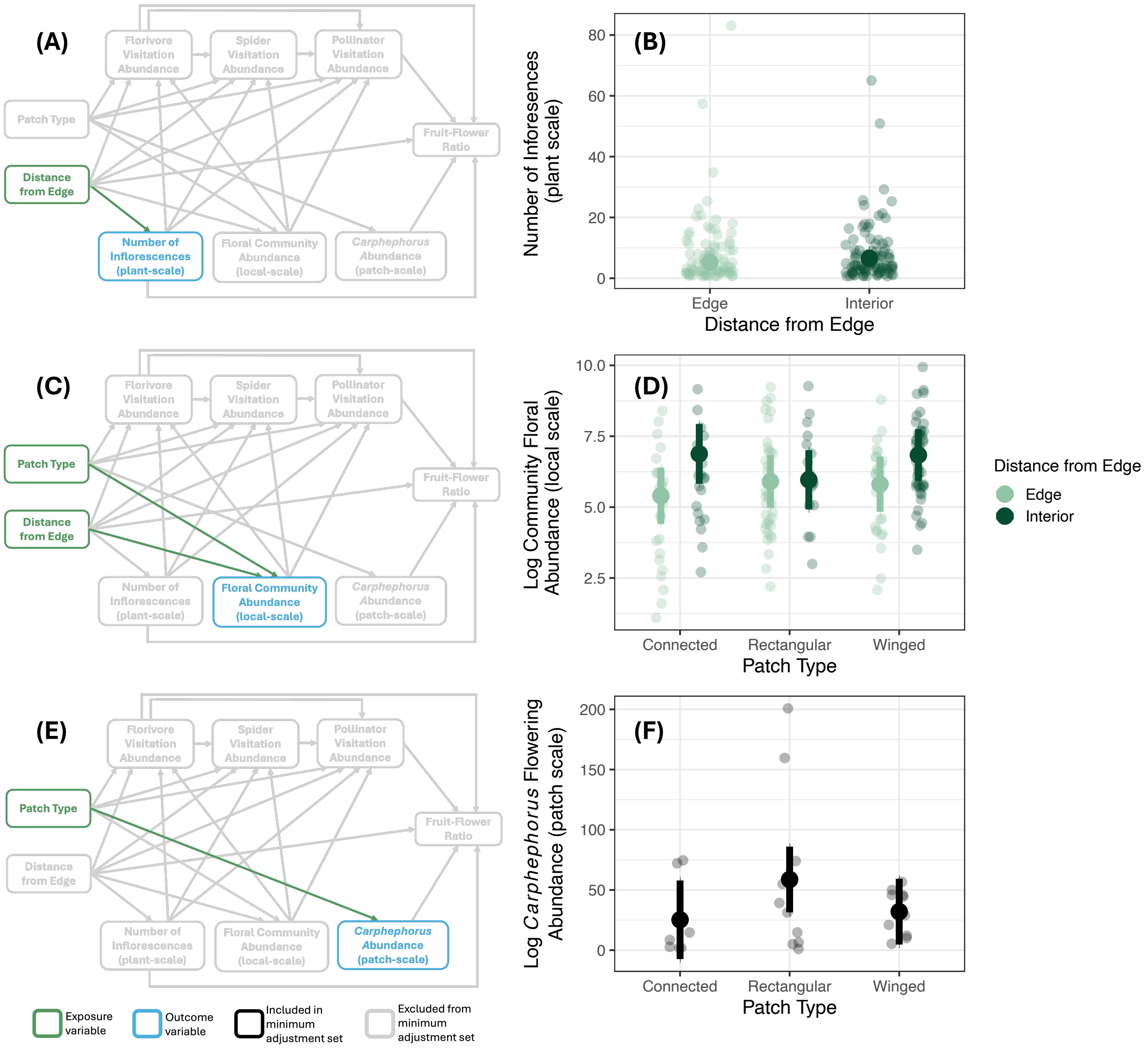

Figure S2. (a, c, e) Directed Acyclic Graphs (DAGs) to determine covariates to estimate the direct effect of distance from an edge or patch type on three measures of floral abundance. Green boxes indicate exposure variable(s) and blue boxes indicate the response variable of interest. (b) The number of focal plant inflorescences (individual plant scale) did not differ at the edge or interior part of the patch. (d) Community floral abundance (local scale) increased at interior parts of the patch, except in rectangular patches. (f) *Carphephorus* flowering abundance (patch scale) did not significantly differ between patch types.

Literature Cited

Balogun, I., O. Eluyeba, O. Adedoja, M. J. Samways, O. Polašek, and T. Kehinde. 2022. Open habitats in a tropical biodiversity hotspot support pollinator diversity in both protected and unprotected areas. Biotropica 54:947–957.

Benoit, A., and S. Kalisz. 2020. Predator Effects on Plant-Pollinator Interactions, Plant Reproduction, Mating Systems, and Evolution. Annual Review of Ecology, Evolution, and Systematics 51.

Boaventura, M. G., N. Villamil, A. L. Teixido, R. Tito, H. L. Vasconcelos, F. A. O. Silveira, and T. Cornelissen. 2022. Revisiting florivory: an integrative review and global patterns of a neglected interaction. New Phytologist 233:132–144.

Bruckman, D., and D. R. Campbell. 2014. Floral neighborhood influences pollinator assemblages and effective pollination in a native plant. Oecologia 176:465–476.

Burt, M. A., and L. A. Brudvig. 2019. Pollen Limitation and Self-Compatibility in Three Pine Savanna Herbs. Southeastern Naturalist 18:405–418.

Camurça, L. M., A. M. M. Santos, C. C. Castro, and A. V. Leite. 2024. Trophic interactions between plants, pollinators, florivores and predators: a global systematic review. Biological Journal of the Linnean Society 141:214–222.

Carper, A. L., L. S. Adler, and R. E. Irwin. 2016. Effects of florivory on plant-pollinator interactions: Implications for male and female components of plant reproduction. American Journal of Botany 103:1061–1070.

Delgado de la flor, Y. A., K. I. Perry, L. M. Collis, P. L. Phelan, and M. M. Gardiner. 2024. Biotic and abiotic factors drive multi-trophic interactions among spiders at different spatial scales in urban greenspaces. Journal of Urban Ecology 10:juae008.

Evans, D. M., N. E. Turley, D. J. Levey, and J. J. Tewksbury. 2012. Habitat patch shape, not corridors, determines herbivory and fruit production of an annual plant. Ecology 93:1016–1025.

Griffin, S. R., and N. M. Haddad. 2021. Connectivity and edge effects increase bee colonization in an experimentally fragmented landscape. Ecography 44:919–927.

Harder, L. D. 1990. Behavioral responses by bumble bees to variation in pollen availability. Oecologia 85:41–47.

Hawn, C. L., J. D. Herrmann, S. R. Griffin, and N. M. Haddad. 2018. Connectivity increases trophic subsidies in fragmented landscapes. Ecology Letters 21:1620–1628.

Hegland, S. J., and L. Boeke. 2006. Relationships between the density and diversity of floral resources and flower visitor activity in a temperate grassland community. Ecological Entomology 31:532–538.

Hulting, K. A., L. A. Brudvig, E. I. Damschen, D. J. Levey, J. Resasco, J. J. Tewksbury, and N. M. Haddad. 2025. Habitat edges decrease plant reproductive output in fragmented landscapes. Journal of Ecology 113:531–541.

Hulting, K. A., L. R. Kemmerling, S. R. Griffin, J. Webb, A. K. Brown, and N. M. Haddad. 2024. Seed mix design and floral resources drive multitrophic interactions in prairie restoration. Journal of Applied Ecology 61:859–868.

Karron, J. D., N. N. Thumser, R. Tucker, and A. J. Hessenauer. 1995. The influence of population density on outcrossing rates in Mimulus ringens. Heredity 75:175–180.

Kilkenny, F. F., and L. F. Galloway. 2008. Reproductive success in varying light environments: direct and indirect effects of light on plants and pollinators. Oecologia 155:247–255.

Knight, T. M. 2003. Floral density, pollen limitation, and reproductive success in Trillium grandiflorum. Oecologia 137:557–563.

Lemoine, N. P., W. A. Drews, D. E. Burkepile, and J. D. Parker. 2013. Increased temperature alters feeding behavior of a generalist herbivore. Oikos 122:1669–1678.

McCall, A. C., and R. E. Irwin. 2006. Florivory: the intersection of pollination and herbivory. Ecology Letters 9:1351–1365.

Mitchell, R. J., J. D. Karron, K. G. Holmquist, and J. M. Bell. 2004. The Influence of Mimulus ringens Floral Display Size on Pollinator Visitation Patterns. Functional Ecology 18:116–124.

Ohashi, K., and T. Yahara. 2002. Visit larger displays but probe proportionally fewer flowers: counterintuitive behaviour of nectar-collecting bumble bees achieves an ideal free distribution. Functional Ecology 16:492–503.

Ruane, L. G., A. T. Rotzin, and P. H. Congleton. 2014. Floral display size, conspecific density and florivory affect fruit set in natural populations of Phlox hirsuta, an endangered species. Annals of Botany 113:887–893.

Su, Q., L. Qi, Y. Yun, W. Zhang, and Y. Peng. 2020. Visual preference of flower-visiting crab spiders (Ebrechtella tricuspidata) for host flowers. Ecological Entomology 45:626–634.

Turley, N. E., J. L. Orrock, J. A. Ledvina, and L. A. Brudvig. 2017. Dispersal and establishment limitation slows plant community recovery in post-agricultural longleaf pine savannas. Journal of Applied Ecology 54:1100–1109.

Uroy, L., A. Ernoult, and C. Mony. 2019. Effect of landscape connectivity on plant communities: a review of response patterns. Landscape Ecology 34:203–225.

Van Geert, A., F. Van Rossum, and L. Triest. 2010. Do linear landscape elements in farmland act as biological corridors for pollen dispersal? Journal of Ecology 98:178–187.

Vrdoljak, S. M., M. J. Samways, and J. P. Simaika. 2016. Pollinator conservation at the local scale: flower density, diversity and community structure increase flower visiting insect activity to mixed floral stands. Journal of Insect Conservation 20:711–721.

Warneke, C. R., T. T. Caughlin, E. I. Damschen, N. M. Haddad, D. J. Levey, and L. A. Brudvig. 2022. Habitat fragmentation alters the distance of abiotic seed dispersal through edge effects and direction of dispersal. Ecology 103:e03586.

Watson, T. L., C. Martel, and G. Arceo-Gómez. 2022. Plant species richness and sunlight exposure increase pollinator attraction to pollinator gardens. Ecosphere 13:e4317.
